# Treatment patterns of prostate cancer with bone metastasis in Beijing: A real‐world study using data from an administrative claims database

**DOI:** 10.1002/pds.4874

**Published:** 2019-08-08

**Authors:** Yinchu Cheng, Lin Zhuo, Yuting Pan, Shengfeng Wang, Jihong Zong, Wentao Sun, Shuangqing Gao, Jian Lu, Siyan Zhan

**Affiliations:** ^1^ Department of Pharmacy Peking University Third Hospital Beijing China; ^2^ Department of Epidemiology and Biostatistics, School of Public Health Peking University Health Science Center Beijing China; ^3^ Research Center of Clinical Epidemiology Peking University Third Hospital Beijing China; ^4^ Department of Epidemiology Bayer HealthCare Pharmaceuticals Inc Whippany New Jersey; ^5^ HEOR and Medical Affairs Bayer Healthcare Co, Ltd Beijing China; ^6^ Department of Data Management Beijing Brainpower Pharmacy Consulting Co Ltd Beijing China; ^7^ Department of Urology Peking University Third Hospital Beijing China

**Keywords:** bone metastasis, claims database, combined androgen blockade, drug utilization, pharmacoepidemiology, prostate cancer, treatment pattern

## Abstract

**Purpose:**

To explore treatment patterns among patients with prostate cancer and bone metastasis and to compare clinical outcomes following use of different hormone therapies including combined androgen blockade (CAB), nonsteroidal antiandrogen (NSAA) monotherapy, and castration monotherapy.

**Methods:**

We conducted a population‐based cohort study using data from the Urban Employee Basic Medical Insurance database (2011‐2014) in Beijing. We identified 475 patients with newly diagnosed bone metastatic prostate cancer with at least one prescription for hormone therapy and described their treatment patterns over a median follow‐up of 20.7 months. Cox proportional hazards model was used to compare time to chemotherapy initiation between patients starting on different hormone therapies.

**Results:**

Hormone therapy and/or bisphosphonate therapy with zoledronic acid were the initial treatments in the majority of patients (87.8%); chemotherapy, radiotherapy, and surgery were usually given later in the treatment pathway. CAB was the most common hormone treatment (73.7%). For time to chemotherapy initiation, hazard ratios (95% confidence intervals) were 2.43 (1.08‐5.44) for NSAA alone vs CAB and 1.29 (0.78‐2.13) for castration alone vs CAB.

**Conclusions:**

Our findings show that while a wide range of therapies are used to treat patients with prostate cancer and bone metastasis in Beijing, hormone therapy and bisphosphonate therapy are the most commonly prescribed, and use of CAB was seen to be advantageous in delaying time to chemotherapy initiation over NSAA monotherapy. Future studies should explore longer‐term treatment patterns, including use of newly approved treatments.

**KEY POINTS**

- We used a claims database to summarize the observed treatment patterns of prostate cancer with bone metastasis in real‐world clinical practice in Beijing; thus, our results will have better generalizability compared with those from clinical trials.
- Hormone therapy and bisphosphonate therapy were the most commonly prescribed treatments for prostate cancer with bone metastasis, in accordance with clinical guidelines.
- Chemotherapy was seldom used as initial therapy, but when used, the most common regimens were docetaxel and estramustine. Radiotherapy and surgery were used less frequently, but when used were often given in a late phase of the treatment pathway.
- Combined androgen blockade potentially has an advantage in delaying chemotherapy initiation over antiandrogen monotherapy. Improper use of antiandrogen monotherapy may need caution though on a minority of patients.
- Further observational studies on Chinese patients with prostate cancer and bone metastasis looking at newer treatments over longer and more recent study periods are needed.

## 1 INTRODUCTION

China has seen a rapid increase in the incidence and prevalence^1^ of prostate cancer in recent decades (ranking sixth in all male cancers), which is likely due to the ageing population and changes in diagnostic practices.^2^, ^3^ In the absence of early diagnosis and primary treatment, prostate cancer commonly progresses to an advanced stage, frequently with bone metastases that have a profound impact on patients' quality of life and place a great burden on healthcare resources.^4^ We have previously shown that bone metastasis in prostate cancer patients in China are common (33.2% in 2011), and higher than in other countries, and related treatment costs have increased.^1^

Continuous androgen deprivation therapy (ADT) as the main hormone therapy is the standard of care for patients with metastatic prostate cancer, providing symptom relief and delaying disease progression.^5^ However, ADT can lead to loss of bone mineral density and an increased risk of osteoporotic fragility fractures. The combined effects of ADT and bone metastasis can result in skeletal complications such as pain, ineffective haematopoiesis, and skeletal‐related events (SREs).^6^ Thus, the management of patients with bone metastatic prostate cancer often requires use of bone‐targeted agents (BTAs) such as bisphosphonates, denosumab, and radium‐233,^4^, ^7^, ^8^, ^9^ as well as palliative radiation, bone surgery, and pain relief. After initial ADT treatment, most patients eventually become unresponsive to castration^10^—metastatic castration‐resistant prostate cancer (mCRPC). First‐line treatment for this patient population has been docetaxel‐based chemotherapy although a few newer agents have been approved in recent years.^11^, ^12^

There are three main types of hormone therapy for patients with prostate cancer.^13^ The first type is antiandrogen alone, usually a nonsteroidal antiandrogen (NSAA) such as bicalutamide, flutamide, and nilutamide. The second type is castration alone, including luteinizing hormone‐releasing hormone (LHRH) agonists or antagonists (ie, medical castration) and bilateral orchiectomy (ie, surgical castration). The third type is medical or surgical castration combined with an NSAA—combined androgen blockade (CAB). Comparative effectiveness studies of the three methods have shown mixed findings, and international guidelines are conflicting.^14^, ^15^, ^16^, ^17^, ^18^, ^19^, ^20^

In China, there are limited data regarding the treatment pathways of patients with prostate cancer and bone metastasis in routine clinical practice, yet this is important knowledge to gain to evaluate whether patients are currently receiving the best available medical treatment. Furthermore, little is also known about the effectiveness of CAB compared with hormone monotherapies. We therefore aimed to explore treatment patterns among this patient population, including frequencies and sequences of therapies used and a comparison of different hormone therapies on the clinical outcomes of time to chemotherapy initiation and SRE occurrence. The study was set in Beijing, which has the nation's most advanced health care service.

## 2 MATERIALS AND METHODS

### 2.1 Study design and data source

We conducted a population‐based cohort study using data from a large medical claims database in Beijing—the Urban Employee Basic Medical Insurance (UEBMI) database. The UEBMI is one of the three main national health insurance schemes in China and is a mandatory program covering urban and retired employees.^21^ The UEBMI contains longitudinal patient data including demographics, clinical diagnoses, and prescriptions for drugs and procedures from all health care institutions in Beijing since 2008.^22^ By the end of 2014, more than 98% of all active and retired urban employees in Beijing were covered by the UEBMI, equivalent to approximately 14 million people. Further details on the database have been published previously.^1^

### 2.2 Study population

We included patients meeting all the following criteria between 1 July 2011 and 31 December 2014: a diagnosis of prostate cancer (International Classification of Diseases [ICD]‐10 code “C61” or associated free text); a diagnosis of bone metastasis (ICD‐10 code “C79.5” or associated free text) on or after the initial diagnosis of prostate cancer; at least one prescription for hormone therapy (antiandrogen, LHRH, oestrogen, or surgical castration). To ensure all patients were incident cases, we applied a 6‐month wash‐out period from 1 January to 30 June 2011. The index date was the date of the first record of bone metastasis. Patients were followed up until the last date of record collection or the end of the study period (31 December 2014), whichever was earlier.

### 2.3 Measurements

We evaluated seven classes of treatments according to the 2014 version of the Chinese Urology Association (CUA) guideline for diagnosis and treatment of prostate cancer and following clinical expert consultation: surgery, radiotherapy, hormone therapy, chemotherapy, bisphosphonate therapy, pain treatment, and traditional Chinese medicine (TCM).^20^ Hormone therapy was classed into three main groups: NSAA alone, castration alone, or CAB. If NSAA treatment period was less than 30 days and the interval between start of LHRH and NSAA treatments was less than 14 days, the patient was included in the castration alone group rather than the CAB group. This was because all patients with planned use of LHRH agonists should be prescribed short‐term antiandrogens to prevent disease flares.^16^

SREs were defined as clinical manifestations of pathological fracture, spinal cord compression, hypercalcemia, or surgery involving bone. Comorbidities were identified using ICD‐10 codes and/or Chinese text in the medical claim records (any time from 1 January 2011 to the index date) and included hypertension, cardiovascular diseases, cerebrovascular disease, type 2 diabetes, and respiratory diseases (asthma and chronic obstructive pulmonary disease). Drug prescriptions were identified using Anatomical Therapeutic Chemical classification codes, and medical procedures were identified using a unified coding system developed by the data owner.

### 2.4 Treatment sequence and clinical outcomes

In addition to identifying the initial treatment prescribed, we described the sequence of treatment over time, in particular noting the first three distinct treatments and the time interval between adjacent treatments. A Sankey plot was produced to visualize the treatment pathways. To compare clinical outcomes after treatment with CAB, NSAA alone, or castration alone, we followed patients from the first date of the respective hormone therapies to identify the occurrence of SREs and the initiation of chemotherapy (as a proxy for disease progression). To do this, we conducted two time‐to‐event analyses, one for each outcome. In these two analyses, patients who did not receive any of the three exposure treatments of interest (eg, patients who only received oestrogens as hormone therapy) and those who already had an outcome event (chemotherapy) before the relevant exposure were excluded. Patients without an outcome of interest were censored when they were last known to be event‐free during the observation period.

### 2.5 Statistical analysis

Patient characteristics and treatments were described using frequency counts and percentages for categorical variables and means with standard deviations or quartiles for continuous variables. Time to chemotherapy/first SRE since first hormone therapy were analysed by multivariate Cox proportional hazards model adjusted for baseline confounders (age, comorbidity, and initial treatment) using a bidirectional stepwise process, with testing for the proportional hazards assumption. Hazard ratios (HRs) with 95% confidence intervals (CIs) were estimated. Direct adjusted survival curves, which display the overall adjusted survival estimates of each treatment, were also produced.^23^, ^24^ A two‐sided *P* value less than.05 was considered strong evidence against the null hypothesis. All analyses were performed using SAS (version 9.4, SAS Institute Inc., Cary, NC, USA).

## 3 RESULTS

### 3.1 Patient characteristics

A total of 475 patients with prostate cancer and bone metastasis were identified (median age was 76 years, interquartile range [IQR], 70‐81 years). The median follow‐up duration from cohort entry was 20.7 months (IQR, 9.7‐32.1 months). Most patients (92.0%, n = 437) had a record of at least one recorded comorbidity; 19.8% (n = 94) of the patients experienced a SRE during follow‐up, with most having five or fewer SRE‐related medical visits (Table 1).

**Table 1:** Demographic and clinical characteristics of patients with prostate cancer and bone metastasis

Characteristic	Number of Patients
	N = 475
	n (%)
Age at index date	
<60	21 (4.42)
60‐69	84 (17.68)
70‐79	220 (46.32)
≥80	150 (31.58)
Calendar year of index date	
2011	102 (21.47)
2012	153 (32.21)
2013	129 (27.16)
2014	91 (19.16)
Comorbidities	
Hypertension	404 (85.05)
Ischaemic heart disease	378 (79.58)
Stroke	280 (58.95)
Type 2 diabetes	206 (43.37)
Asthma or COPD	199 (41.89)
Number of comorbidities	
0	38 (8.00)
1	45 (9.47)
2	75 (15.79)
≥3	317 (66.74)
Number of SRE‐related visits	
0	381 (80.21)
1‐5	88 (18.53)
>5	6 (1.26)
Cancer treatment	
Hormone therapy	475 (100)
Castration alone (orchiectomy or LHRH‐based)^a^	94 (19.79)
NSAA alone	25 (5.26)
CAB	350 (73.68)
Oestrogen	99 (20.84)
Radiotherapy	119 (25.05)
Surgery	82 (17.26)
Radical prostatectomy	59 (12.42)
Surgery involving bone	27 (5.68)
Chemotherapy	175 (36.84)
Bisphosphonate therapy	360 (75.79)
Pain treatment	433 (91.16)
TCM treatment	431 (90.74)

*Note*. Categories are not mutually exclusive; hence, percentages may sum over 100.

Abbreviations: CAB, combined androgen blockade; COPD, chronic obstructive pulmonary disease; LHRH, luteinizing hormone‐releasing hormone; NSAA, nonsteroidal antiandrogen; SRE, skeletal‐related event; TCM, traditional Chinese medicine.

a All patients with planned use of LHRH agonists should have antiandrogens for disease flares.

### 3.2 Treatments at any time during follow‐up

Of the three classes of hormone therapy, most patients were treated with CAB (73.7%), 19.8% were treated with castration alone and only 5.3% were treated with NSAA alone. Approximately 7.6% of patients were surgically castrated (bilateral orchiectomy). Oestrogens were used in 20.8% of patients, of whom a few (n = 6) received oestrogens solely as hormone therapy. The majority of patients received pain treatment (91.2%) and TCM (90.8%). Other commonly received treatments were bisphosphonate therapy (75.8%) and chemotherapy (36.8%). The main types of surgery performed were radical prostatectomy and surgery involving bone (Table 1).

### 3.3 Prostate cancer‐related hospital visits

A total of 16 205 prostate cancer‐related hospital visits were made by the 475 patients during the observation period, most of which took place in tertiary hospitals, urology departments, and outpatient clinics. The number of hospital visits for each type of cancer treatment stratified by hospital tier, department, and visit type (inpatient/outpatient) is shown in Table 2.

**Table 2:** Distribution of prostate cancer‐related hospital visits for specific cancer treatment among patients with prostate cancer and bone metastasis, stratified by hospital tier, department, and type (inpatient/outpatient)

	Surgery N = 113 n (%)	Radiotherapy N = 161 n (%)	Hormone Therapy N = 7550 n (%)	Chemotherapy N = 757 n (%)	Bisphosphonate Therapy N = 2641 n (%)	Pain Treatment^a^ N = 2393 n (%)	TCM N = 5071 n (%)	Total Visits N = 16 205 n (%)
Hospital tier^b^								
Tertiary	107 (94.7)	158 (98.1)	7062 (93.5)	678 (89.6)	2355 (89.2)	2005 (83.8)	4189 (82.6)	14 594 (90.1)
Secondary	5 (4.4)	3 (1.9)	488 (6.5)	72 (9.5)	283 (10.7)	361 (15.1)	679 (13.4)	1299 (8.0)
Primary	1 (0.9)	0 (0.0)	0 (0.0)	7 (0.9)	3 (0.1)	27 (1.1)	203 (4.0)	312 (1.9)
Department								
Urology	53 (46.9)	65 (40.4)	5342 (70.8)	285 (37.7)	1224 (46.4)	715 (29.9)	1547 (30.5)	9265 (57.2)
Oncology	3 (2.7)	25 (15.5)	645 (8.5)	180 (23.8)	474 (18.0)	468 (19.6)	944 (18.7)	1824 (11.3)
Surgery	32 (28.3)	22 (13.7)	917 (12.2)	153 (20.2)	342 (13.0)	452 (18.9)	934 (18.4)	1629 (10.1)
Internal medicine	0 (0.0)	4 (2.5)	327 (4.3)	46 (6.1)	181 (6.9)	377 (15.8)	676 (13.3)	1182 (7.3)
TCM	3 (2.7)	2 (1.2)	41 (0.5)	1 (0.1)	52 (2.0)	64 (2.7)	437 (8.6)	496 (3.1)
Radiology	0 (0.0)	26 (16.2)	90 (1.2)	19 (2.5)	57 (2.2)	36 (1.5)	45 (0.9)	390 (2.4)
Orthopaedics	16 (14.2)	0 (0.0)	43 (0.60)	41 (5.4)	173 (6.6)	67 (2.8)	148 (2.9)	355 (2.2)
Emergency	0 (0.0)	0 (0.0)	9 (0.1)	4 (0.5)	3 (0.1)	60 (2.5)	83 (1.6)	333 (2.1)
General practice	0 (0.0)	0 (0.0)	20 (0.3)	2 (0.3)	15 (0.6)	30 (1.3)	84 (1.7)	222 (1.4)
Other	6 (5.3)	17 (10.6)	116 (1.5)	26 (3.4)	120 (4.5)	124 (5.2)	173 (3.4)	509 (3.1)
Type								
Outpatient	9 (8.0)	47 (29.2)	5202 (68.9)	206 (27.2)	1498 (56.7)	1252 (52.3)	3023 (59.6)	13 038 (80.5)
Inpatient	104 (92.0)	114 (70.8)	2348 (31.1)	551 (72.8)	1143 (43.3)	1141 (47.7)	2048 (40.4)	3167 (19.5)
Number of visits per capita	1.4	1.4	15.9	4.3	7.3	6.6	11.8	34.1

*Note*. Percentages may not add up to 100 because of rounding. Sums of row numbers are not equal to total visits in each row because of overlap among groups.

Abbreviations: TCM, traditional Chinese medicine.

a Includes opioids, nonsteroidal anti‐inflammatory drugs, antidepressants, and anticonvulsion drugs.

b Tertiary is the highest tier.

### 3.4 Commonly used drugs

Table 3 shows the numbers of patients prescribed specific hormone therapy and chemotherapy agents, and the number of medical/hospital visits made for use of each of these therapies. Among the hormone therapies, NSAAs (predominantly bicalutamide followed by flutamide) and LHRHs (mostly leuprorelin or goserelin followed by triptorelin) were the most commonly prescribed drugs. Oestrogens (mainly megestrol) were less frequently prescribed. Among chemotherapy drugs, docetaxel was the most commonly prescribed, followed by estramustine, platinum‐based agents, and fluorouracil.

**Table 3:** Commonly used hormone therapy and chemotherapy drugs among patients with prostate cancer and bone metastasis

Drugs	Number of Patients (%)	Number of Visits (%)	Number of Visits Per Capita
Hormone therapy	475 (100.0)	7550 (100.0)	15.9
Antiandrogen			
Bicalutamide	378 (79.6)	3355 (44.4)	8.9
Flutamide	125 (26.3)	539 (7.1)	4.3
LHRH			
Leuprorelin	285 (60.0)	2525 (33.4)	8.9
Goserelin	268 (56.4)	2371 (31.4)	8.8
Triptorelin	147 (30.9)	828 (11.0)	5.6
Oestrogen			
Megestrol	75 (15.8)	206 (2.7)	2.7
Oestradiol valerate	25 (5.3)	108 (1.4)	4.3
Medroxyprogesterone	14 (2.9)	22 (0.3)	1.6
Chemotherapy	175 (100.0)	757 (100.0)	4.3
Docetaxel	74 (42.3)	361 (47.7)	4.9
Estramustine	30 (17.1)	73 (9.6)	2.4
Platinum‐based	26 (14.9)	70 (9.2)	2.7
Fluorouracil	21 (12.0)	71 (9.4)	3.4
Anthracyclines	17 (9.7)	40 (5.3)	2.4
Gemcitabine	8 (4.6)	28 (3.7)	3.5
Camptothecin	6 (3.4)	15 (2.0)	2.5
Cyclophosphamide	5 (2.9)	21 (2.8)	4.2

*Note*. Percentages may sum over 100 because overlap of different drugs. Less commonly prescribed drugs are not shown in the table.

### 3.5 Treatment pathways

**Figure 1:**
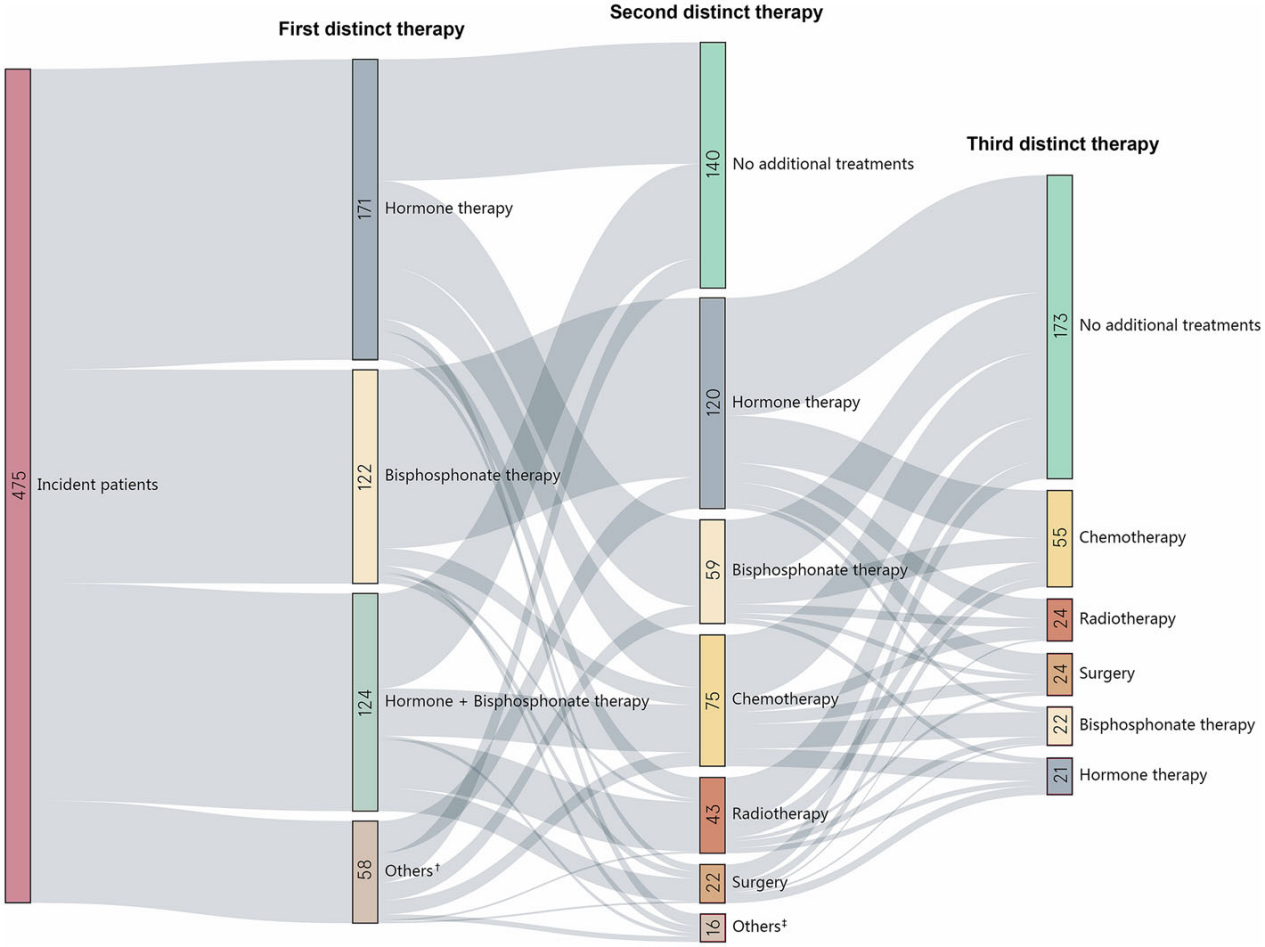
Common treatment pathways among incident patients with prostate cancer and bone metastasis. ^†^Treatments included chemotherapy, surgery or radiotherapy, alone or in combination with other therapies. ^‡^Combination therapies. Future treatments were not looked at for this group. Data shown are first three distinct treatments in the sequence of initiation (from left to right). Size of each box (with the number on it) and thickness of the connecting lines indicate the quantity of patients on the relevant treatment pathway. Adjuvant therapies (pain treatment and traditional Chinese medicine) are not shown in the figure for simplicity [Colour figure can be viewed at http://wileyonlinelibrary.com]

Treatment pathways over time are illustrated in Figure 1. A total of 102 different treatment pathways were identified in these patients. Most patients (87.8%, n = 417) started with hormone therapy and/or bisphosphonate therapy, 27 (5.7%) started with treatments involving chemotherapy, 21 (4.4%) with treatments involving radiotherapy, and 15 (3.2%) with treatments involving surgery. Chemotherapy was mostly used as a second (15.8%, n = 75) or third (11.6%, n = 55) therapy, radiotherapy as a second (9.1%, n = 43) therapy, and surgery as third (5.1%, n = 24) therapy. The median time between the initiation of adjacent treatments in each pathway ranged from 23 to 570 days. The weighted average of the median time from start of previous treatment to hormone therapy was 49 days, to bisphosphonate therapy was 90 days, to chemotherapy was 194 days, to radiotherapy was 171 days, and to surgery was 226 days.

### 3.6 Time to initiation of chemotherapy and first SRE

**Figure 2:**
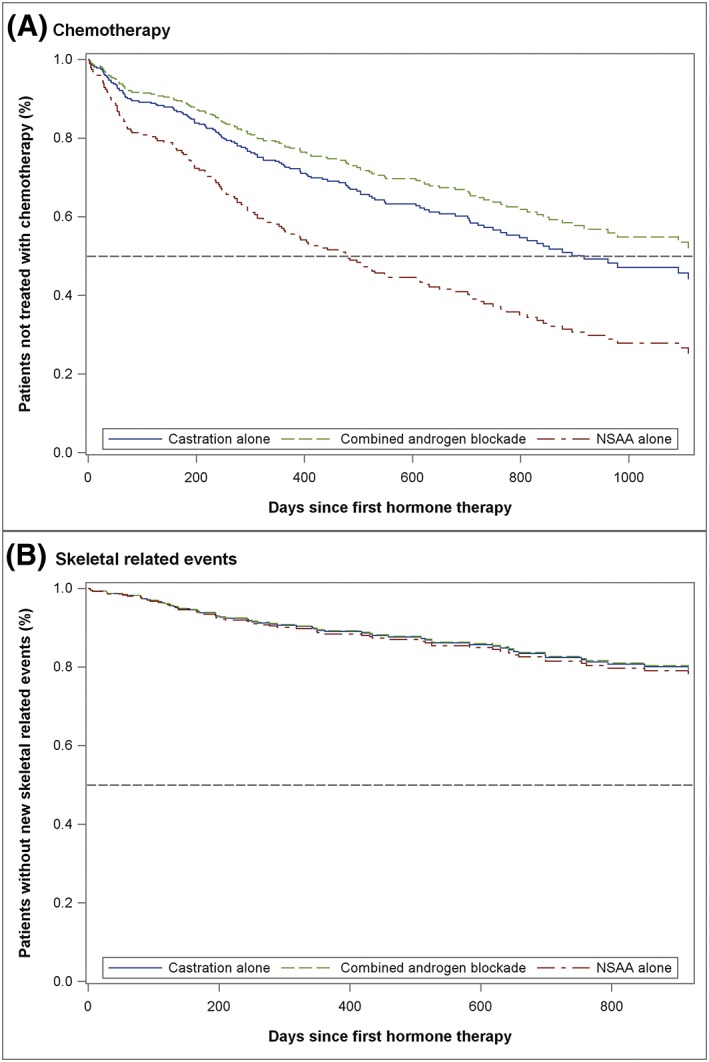
Direct adjusted survival curves for A, time to chemotherapy initiation and B, time to first skeletal‐related event, according to the initial hormone therapy prescribed. Each curve shows the average of the predicted survival estimates generated from adjusted Cox regression for all the patients in the relevant hormone treatment group. The dashed line indicates that the outcome of interest has occurred in half of the patients. NSAA, nonsteroidal antiandrogen [Colour figure can be viewed at http://wileyonlinelibrary.com]

Adjusted HRs and median progression time from first hormone therapy to initiation of chemotherapy/first SRE are presented in Table 4. Direct adjusted survival curves are shown in Figure 2. After adjusting for covariates, patients treated with NSAA alone had more than double the likelihood of subsequently receiving chemotherapy compared with those treated with CAB (HR, 2.43; 95% CI, 1.08‐5.44; *P* = .031), while no significant difference was found between other hormone therapy treatment groups for chemotherapy initiation. In the time to first SRE analysis, the median progression time to SRE was not reached in each group and there was no evidence of difference in time to first SRE between hormone groups.

**Table 4:** Adjusted HRs (95% CI) for time to chemotherapy initiation and time to first SRE after hormone therapy, in patients with prostate cancer and bone metastasis

Outcome	Median Progression Time to Events (No. of Events/No. of Patients)			HR (95% CI)	*P* Value
	CAB	NSAA alone	Castration alone		
Initiation of chemotherapy (n = 431)^a^	NR (109/325)	477 days (7/22)	895 days (19/84)		
NSAA alone vs CAB (reference)				2.43 (1.08‐5.44)	.031
Castration alone vs CAB (reference)				1.29 (0.78‐2.13)	.326
NSAA alone vs castration alone (reference)				1.88 (0.76‐4.67)	.171
First SRE (n = 469)^b^	NR (52/350)	NR (2/25)	NR (8/94)		
NSAA alone vs CAB (reference)				1.01 (0.24‐4.27)	.993
Castration alone vs CAB (reference)				1.08 (0.49‐2.36)	.853
NSAA alone vs castration alone (reference)				0.94 (0.19‐4.60)	.934

*Note*. Covariates to be included in the final model were selected by a stepwise cox regression with significance levels of.25 for entering effects and.15 for removing effects.

Abbreviations: CAB, combined androgen blockade; CI, confidence interval; HR, hazard ratio; NSAA nonsteroidal antiandrogen; NR, not reached during the observation period; SRE, skeletal‐related event; TCM, traditional Chinese medicine.

a Covariates including age, pain treatment, TCM treatment, and comorbidity of ischemic heart disease at baseline were adjusted for in the final model.

b Covariates including age, previous SREs, TCM treatment, bisphosphonate treatment, and comorbidity of hypertension at baseline were adjusted for in the final model.

## 4 DISCUSSION

In this population‐based cohort study, we have shown that hormone therapy and bisphosphonate therapy with zoledronic acid are the most commonly used for patients in Beijing with prostate cancer and bone metastasis, with the majority of patients starting treatment with one or both of these therapies, in accordance with current guidelines.^19^, ^25^ We have also shown that time to chemotherapy initiation was longer among patients receiving CAB vs NSAA alone. There was no strong evidence of a difference in time to chemotherapy initiation between patients receiving CAB/NSAA vs castration monotherapy.

CAB with bicalutamide was the most commonly used hormone therapy among patients in our study, in line with the CUA 2014 guideline.^20^ Approximately one in 20 patients were prescribed NSAA monotherapy despite being recommended in the CUA guidelines as a treatment option only for advanced localized prostate cancer and not metastatic disease. In an administrative claims database study in the United States, Flaig et al^26^ reported that 14% of patients with bone metastatic hormone‐sensitive prostate cancer (mHSPC) received prostate cancer drug only and no castration. Both physician and patient factors could account for the prescribing of NSAA monotherapy, including lack of knowledge of the most recent treatment guidelines especially among physicians in primary care, and lesser acceptance of LHRH injection therapy with certain patient conditions like skin disorders and the possibility of related adverse events, over oral administration of NSAA; 93.5% of patients received castration in our study, the proportion is higher than that reported by Flaig et al (65%). Among them, medical castration (85.9%) was a more common form of castration than surgery (7.6%), though they appear equally effective with orchiectomy proved even safer recently.^27^, ^28^ Possible reason is that medical castration is potentially reversible and avoids the physical and psychological discomfort associated with orchiectomy.^29^

Bone‐targeting agents reduce bone reabsorption by primarily targeting osteoclasts.^4^ They are important in the treatment of patients with bone metastatic prostate cancer, and if not used, approximately half of patients will experience one or more SREs within 2 years.^30^ Data from clinical trials suggest that conventional bone‐targeting treatments, such as bisphosphonate therapy with zoledronic acid, which was prescribed to most patients in our study, does not increase overall survival.^9^, ^30^ Radium‐233—one of several newer agents available—has, however, been shown to prolong survival in addition to reducing symptomatic bone complications.^31^

Forty‐two percent of patients in our study were prescribed chemotherapy, which is very similar to that reported in a claims database study in the United States (45.4%).^32^ Docetaxel is the standard of care in patient with CRPC and was the most commonly used chemotherapy drug in our study cohort, followed by estramustine, which is no longer recommended by the American guideline^19^ but still retained in the Chinese guidelines.^20^, ^25^ Since 2015, the use of chemotherapy together with ADT as initial therapy for mHSPC patients has been supported by clinical guidelines.^5^, ^33^ In our study, we found that chemotherapy was rarely prescribed as the initial therapy and therefore was likely a sign of disease progression, as used as a secondary endpoint in a recent clinical trial.^11^

Radiotherapy and surgery were less frequently used and usually given late in the treatment pathway. Similar treatment patterns were observed by Seal et al in a hospital database study.^34^ However, for the specific drugs used in each class of treatment, patterns found in our study differed from that in a US population,^35^ mainly because newer agents such as abiraterone, denosumab, radium‐233, and sipuleucel‐T were neither approved nor reimbursed in China during the study period and so were not captured in the database.

Studies comparing CAB, NSAA alone, and castration alone have shown mixed findings. In advanced prostate cancer patients, CAB was reported to have an survival advantage than castration alone,^14^, ^18^, ^36^, ^37^ and the latter was reported to be more effective than NSAA alone in a Cochrane review.^16^ However, an observational study found no significant difference of overall survival and adverse events between bicalutamide 150 mg and CAB in patients with locally advanced prostate cancer,^15^ yet other studies have suggested greater toxicity and decreased quality of life among patients receiving CAB compared with NSAA alone.^38^, ^39^ Current clinical guidelines differ in respect to their recommendations on use of CAB for prostate cancer patients with bone metastasis. The American Society of Clinical Oncology (ASCO), CUA, and European Association of Urology (EAU) state that CAB has a survival benefit over castration alone, while National Comprehensive Cancer Network (NCCN) states that more evidence is required, especially from prospective randomized trials, to be confident about a survival advantage with CAB. According to ASCO and CUA, NSAA alone may be discussed as an alternative in patients at certain stage, while EAU is more neutral to its use and NCCN does not recommend its use.^5^, ^17^, ^19^, ^20^ The results of our study moderately add to the evidence favouring CAB over NSAA alone but not over castration alone on delaying chemotherapy initiation in Chinese bone metastatic prostate cancer patients. We did not find any differences in occurrence of SREs between CAB and hormone monotherapies, although it should be noted that it is BTAs rather than hormone therapies that specifically aim to reduce these outcomes.

Our study has several strengths. To our knowledge, it is the first to look at treatment patterns among patients with prostate cancer and bone metastasis in China and thus provides valuable clinically important information describing the medical management of these patients. Without data such as ours to use as a benchmark, it would be difficult to describe use and effectiveness of newer approved medications as they become approved and prescribed in China, through both future descriptive and analytical epidemiological studies. We used a large database representative of urban employees in Beijing enabling a reasonable sample size to be acquired. Our study also has some limitations. Firstly, the UEBMI database does not provide results of mortality data or detailed clinical and pathologic information; hence, we were unable to directly identify patients with mCRPC, and we used time to chemotherapy initiation and first SRE as surrogate outcome measures. Secondly, the sample size might still be insufficient to detect small to moderate differences, and the observation period was short leading to many censored observations in the survival analysis especially for the SRE endpoint, which to some extent undermines the observed results. Finally, Beijing is one of the few first tier cities in China with better health care resources; therefore, treatment patterns identified in this study cannot necessarily be generalized to other regions of China.

## 5 CONCLUSIONS

Our findings show that while a wide range of therapies are used to treat patients with prostate cancer and bone metastasis in Beijing, hormone therapy and bisphosphonate therapy remain the cornerstone of treatment, with CAB potentially having an advantage in delaying chemotherapy initiation over NSAA monotherapy. Improper use of NSAA alone in this patient population, though found only in isolated cases, should be avoided. Future studies among patients with prostate cancer and bone metastasis in China with longer follow‐up periods are needed to revisit this topic especially as newly approved treatments are more prescribed. Evaluation of physician knowledge of current clinical guidelines and patient adherence in this area would also be a valuable line of research.

## ETHICS STATEMENT

This study was approved by the Ethical Review Board of Peking University Health Science Center (No. IRB00001052‐16027‐Exempt), and informed consent of participant was exempted.

## CONFLICT OF INTEREST

J.Z. is an employee of Bayer HealthCare Pharmaceuticals Inc. W.S. is an employee of Bayer Healthcare Co, Ltd. S.G. is an employee of Beijing Brainpower Pharmacy Consulting Co Ltd. All authors declare no potential conflict of interest.

## AUTHOR CONTRIBUTIONS

Y.C., L.Z., Y.P., S.Z., and J.L. participated in the study design. Y.C. and L.Z. performed the statistical analysis. Y.C. drafted the manuscript. S.W., W.S., and J.Z. revised the manuscript. J.L. provided clinical support. S.G. contributed to data acquisition. S.Z. and J.L. got the funding. All authors read and approved the final manuscript.
